# CAM Use and Suggestions for Medical Care of Senior Citizens: A Qualitative Study Using the World Café Method

**DOI:** 10.1155/2013/951245

**Published:** 2013-08-18

**Authors:** B. Stöckigt, M. Teut, C. M. Witt

**Affiliations:** Institute for Social Medicine, Epidemiology, and Health Economics, Charité, Universitätsmedizin Berlin, 10117 Berlin, Germany

## Abstract

*Background*. Little data exists concerning the reasons for using complementary and alternative (CAM) therapies by seniors. Therefore, the aim of this study is to learn about motives of German seniors for using CAM therapies and their wishes for health care in general. *Methods*. One focus group and one “World Café” following a semistructured interview guide were conducted. All discussions were recorded digitally, transcribed, and analyzed according to Qualitative Content Analysis using the software MAXQDA. *Results*. In total 21 seniors participated (eighteen female, three male, mean age 72.5 ± 4.3 years). Most of the participants had lifelong experiences with medicinal herbs and home remedies due to unavailable conventional care during childhood. Also important for them were nutrition and exercise. These methods were often used as self-care to enhance wellbeing, to prevent and to cure illnesses. The participants would welcome an integration of CAM in health care services. They asked especially for more empathic physicians who are better trained in CAM and respect their experiences. *Conclusion*. The importance of life experience in regard to health care by senior can be seen as a resource. Qualitative studies investigating physician-patient relationships and intergenerational aspects in CAM use could be of interest for further studies.

## 1. Background

There is minimal available data in Germany regarding the use and the reasons for the use of complementary and alternative (CAM) therapies by senior citizens. Yet the Allensbach inquiry [1] highlights that the number of the users of CAM has risen at the age of 60 years and older from 61% (1970) to 80% (2002) without specifying therapies and reasons.

In the USA, several surveys were carried out with elderly patients. A survey by Cheung et al. (2007) with 1200 participants over 65 years of age showed that around two thirds (62.9%) applied one or more (on average three) complementary and alternative medical treatments at the same time. Eighty percent of the users reported a high satisfaction [2]. The maintenance of health and the treatment of health complaints such as arthritis and chronic pain were given as reasons for the use of CAM. Supplements, for example, vitamins and herbs, prayer meditation, and chiropractic were applied predominantly. Motives of the use were to reduce symptoms of disease and the desire for personal control of health. In the survey by Ness et al. 88% of the over 65-year olds applied CAM and showed that CAM usage increased with age. The most often used CAM treatments were diet and chiropractic manipulation/care. Men applied the CAM treatments less often than women. The majority of the senior citizens did not inform their doctors about the CAM use and paid for the treatments largely out of pocket [3]. Studies show that potentially inappropriate medication for elderly patients in conventional medicine can be dangerous [4–7]. The lack of integration of CAM and conventional medicine and not informing doctors about CAM use may pose further danger for health [2].

The aim of this study is to learn about reasons and motives of German seniors for using complementary and alternative (CAM) therapies and their ideals for optimum health care delivery.

## 2. Methods

### 2.1. Study Design

We used focus discussion groups and the discourse method “World Café.” Both are used in collaborative dialogues wherein knowledge is gathered and shared. Whereas focus groups are suitable for small groups, the “World Café” method is a simple, effective, and flexible format for hosting large group dialogue. “World Café” is especially applied in group settings where community participation and community organization are required and makes use of the creative atmosphere which often emerges at a café [8, 9]. In this study participants were asked to sit at one of three tables and discuss one of the predefined questions for about 20 minutes. The discussions at each table were moderated by one researcher of the Institute of Social Medicine who was trained in moderation and the “World Café” method. At the end of each round, the moderator remained at each table as a host, while the participants moved to different tables and formed new groups. After three rounds, the whole group gathered in an auditorium for sharing collective discoveries and a closing discussion. In contrast to the “World Café” was the focus group led by one interviewer/moderator all the time; there were no breaks in between and no changes in the group setting. The predefined questions were the same in the focus group and World Café.

Topic 1: therapies of CAM:

 what kind of drugs and therapies do you use and for what kind of problems?

Topic 2: integration in health care:

 how do you combine CAM and your normal health care?

Topic 3: suggestions for an ideal medical care:

 what does good health care look like for you?
 how far should CAM be integrated?

### 2.2. Data Collection and Sample

We developed a list of relevant meeting places for seniors in Berlin and contacted them in a consecutive order. Some of them had no interest in the study or had only irregular meetings of small groups. We included the first one that was interested to participate and had regular meetings. In this neighborhood centre seniors meet every second week for breakfast with an additional cultural event. In general more women attend these meetings, and although male and female seniors were invited to the “World Café,” only women came. In addition one focus group with three male seniors who were recruited over the integrative medicine outpatient clinic of the Charité was conducted for men's perspective at the Institute of Social Medicine. Prior to the “World Café” sessions and the focus group meeting interviewer and moderators did not know any of the participants of the study. 

The discussion was digitally recorded and then transcribed verbatim. Additionally the participants completed an anonymous questionnaire about individual sociodemographic aspects and CAM use.

### 2.3. Analysis

The discussions were analyzed according to Qualitative Content Analysis [10] using the computer program MAXQDA by the first author who is a trained and experienced qualitative researcher. Categories were built deductively according to the themes of the interview guide and inductively according to the topics which came up in the discussions. For quality assurance the analysis was discussed with other experienced qualitative researchers in the qualitative working group at the Charité (Institute of Social Medicine and Berlin School of Public Health). In this group transcripts and analysis are discussed to reflect the influence of the opinions of each researcher on the analysis and to ensure intersubjectivity. 

### 2.4. Ethical Issues

Participants provided informed consent and the study was approved by the Ethics Committee of Charité Universitätsmedizin Berlin (EA1/087/11).

## 3. Results

We included 21 participants (18 women in the World Café and 3 men in the focus group) with a mean age of 72.5 (SD = 4.3) years (demographic characteristics are shown in Table 1). Almost half of the participants (48%) have used CAM before (see Table 2 for details on CAM use). The German term “Komplementärmedizin” (complementary medicine) was not known by most of the participants and never used throughout the discussions. Instead they talked about *“natural remedies” *(female senior (FS) 23), products from *“nature”* (FS 2), and *“alternative medicine”* (FS 2). For summing up different therapies the term CAM will still be used in this paper. 

The most important theme, which came up in the discussions, was medical plants and the so-called “home remedies” being part of the participants' life experience dating back to childhood where conventional medicine was not available. Relating to this a natural and pragmatic attitude to health care became apparent. The participants used methods they knew for decades, had positive experiences with, had easy access to, and felt natural to them. They highlighted herbal medicines, home remedies, nutrition, and exercise. Related to the usage was the importance of taking one's own initiative. Participants wanted to get information about treatment options and self-care options from their doctors but also wanted that their lifelong experiences were respected. All participants complained about the high-tech-oriented health care system and asked especially for more empathy in the doctor-patient relationship. Furthermore they would appreciate the integration of CAM into the health care and asked for doctors that are more knowledgeable in CAM. Additionally specific issues and worries of older adults were discussed, such as possible dependency on care and being perceived as a burden in society.

### 3.1. The Importance of Life Experiences

Most of the participants had knowledge and experiences about medicinal plants and especially home remedies dating back to the days of their childhood, which were available in time of and after war in contrast to a less available and less developed conventional medicine. For example, most participants remembered the time before the regular use of antibiotics.

 FS 12: *“As a child I got lime-tree tea, that was post war period (…) there were somehow or other no drugs, they had to take simply from nature.” *

There were extensive discussions about these experiences. Some therapies were perceived as very helpful and were still used today, for example, wraps, whereas others were seen as uncomfortable and the participants appreciated the replacement with conventional drugs.

 FS 12: *“Some things are also out-dated, I got cod liver oil and sun lamp as a child (…) With the sunlamp I burnt my hand, that was easier for my daughter, [with] a small [Vitamin D-] pill (…) I don't like going back to cod liver oil and sunlamp.” *
 FS 13: *“Children were tortured with this in the past.” *
 FS 11: *“There was not much more (…) that was probably the only remedy, this awful, cod liver oil, this fatty one.” *

Subsequently, they experienced the progress in conventional medicine and the neglect of home remedies and use of medicinal plants. The current positive trend in society towards CAM was welcomed, although with some amusement, because it was perceived to be marketed as something new.

 FS 13: *“It is also right that the trend is moving to it again, that one is again more (…) trying out also with natural remedies.” *
 FS 11: *“But they claim if this would be a new invention, that what I always wonder about.” *

### 3.2. CAM Use: Frequent, Diverse, and Pragmatic

The majority used CAM methods regularly for various physical and mental problems, as well as prevention of illnesses and to avoid dependency on care. Herbal medicine was often favored compared to conventional treatment because of perceived threats such as side effects from conventional drugs. Yet there were extremely controversial ideas about the potential of CAM.

 FS 22: *“Basically it is logical that a natural thing helps better than chemistry.” *
 FS 13: *“Nature does not always help.” *
 FS 2: *“In nature you find also some poisons.” *

Some participants (especially women) were interested in the holistic approach, wherein the interaction between mind and body was seen to enhance general wellbeing.

 FS 22: *“Because every physical illness has something to do with the soul, (…) always.” *

In general the participants had a pragmatic approach and used those methods they were familiar with for a long time, had easy access to, had positive experiences with, and that felt natural to them. Relating to this the most mentioned therapies were herbal medicine, the so-called home remedies, nutrition, and exercise (Table 2).

The spectrum of used herbs, the application, and the knowledge about it varied. The most popular were sage, arnica and chamomile. There were many positive experiences reported, but also various limitations, for example, in taste, long duration until any effects occur, and possible side effects.

 FS 3: *“I kind of fell in love with chamomile (…) I almost do everything with chamomile.” *
 FS 1: *“I made a compress with arnica (…) and got a terrible allergy.” *

There were intense discussions about home remedies. Much attention was given to wraps of all kinds, warm or cold, with additives or without especially in the World Café of the female seniors. Despite the reported effort in their application, they were used for various inflammations (arthritis, bronchitis) and the participants welcomed that occasionally wraps were used even in conventional hospitals.

 FS 13: *“…I always do it before going to sleep (…) and in the morning there is a big mess (…) in the morning the fresh curd cheese is dry which takes out the inflammation, but it is so helpful (…) it helps incredibly.” *

The participants, especially all men, showed great concern about food regarding their health. Still, especially the male seniors agreed that one should not exaggerate, even if this contrasted their wives opinions. Again often they referred to life experiences and traditions.

 MS 31: *“Once a week, there has to be fish; that one knows from childhood.” *

Repeatedly there were discussions regarding whether exercise belongs to the so-called CAM or not. But more important than the definition was the huge importance of exercise for physical and mental health and general wellbeing especially in older age for all participants. One participant with arthritis (FS 15) reported with pride that she could avoid a knee operation due to regular exercise. It was noticeable how many different kinds of exercise were known and said to be performed (walking, bicycle riding, qigong, water gymnastics, and more). Relaxation techniques were only mentioned by women and especially used in combination with exercise. Two users (FS 3, FS 5) were especially delighted by the simplicity in use of breathing exercises and their various effects like wellbeing, calmness, and feelings of happiness.

 MS 31: *“I also need my exercise and move where and as often as possible (…) it is good for me, that I notice. Fresh air is important anyway.” *
 FS 2: *“After gymnastics we always do it [relaxation], that is very pleasant, the whole body calms down again fantastically (…) and everything gets relaxed nicely.” *

Lesser mentioned and discussed methods were, for example, homeopathy and acupuncture; those were partly seen with curiosity, partly with skepticism, and even cynicism. The effects were judged from a good effect to no effect at all. The effects of homeopathy, for instance, were described as small, soft, and not sufficient. Instead, the efforts a homeopathic treatment required were seen partly as annoying. For most of the elderly who had experiences with acupuncture the effects were not convincing or/and they suffered from side effects like dizziness or tiredness. However, some reported good effects and showed an interest in acupuncture treatment.

 FS 21: *“Years ago I went to a homoeopathist. (…) That was also a flop. (…) What one was not allowed to do anymore! What was all forbidden, one was only considering, not the tooth paste…” *
 FS 23: *“I was always very tired [after acupuncture treatment], I had to lay down after coming home.” *
 FS 22: *“I was always beautifully relaxed after getting up [after acupuncture treatment] and then I felt first of all pretty good for some days.” *

### 3.3. Integration of CAM in Health Care

In all groups' discussions about integration started with complaints about the current health care system. It was highlighted that physicians have not enough time, that empathy with and interest in the patient are lacking.

 FS 3: *“My family doctor does not listen to my lungs anymore, she sends me to the laboratory…” *
 FS 4: *“at the orthopedists (…) that was really like in military, I only had to say yes und no (…) they did not explain anything (…) and I don't tell anything anymore either (…) also to my family doctor, (…) they are always in a hurry (…) I get the feeling they do not want to talk to you.” *

Many participants used some type of CAM therapies, but few did inform their doctors about it. Besides lack of time and interest, it was mentioned that it could not be taken for granted that physicians accept its use. Furthermore, some participants had doubts about the level of knowledge of their doctors in CAM. According to the participants lack of acceptance and lack of knowledge interact.

 FS 4: *“I do not know, if he is trained properly, if he knows enough about it [CAM].” *
 FS 25: *“Acceptance comes only from experience (…) I cannot (…) accept something that I don't know.” *

Therefore one's own initiative was very important concerning health care in general. It was seen as necessary to receive useful information and individually fitting therapies and to find *“good”* physicians who also encouraged one's own initiative. Concerning CAM participants' initiatives included getting books, asking professionals, attending talks and courses, and getting information from the social surrounding. Most of the participants reported that over time they had found doctors they trusted and even consulted especially doctors who had an extra qualification in CAM. Still the participants often first tried to treat their symptoms themselves with, for example, home remedies. If this did not work or if they suffered from more serious diseases, they consulted a doctor for conventional medicine treatment.

 FS 13: *“Then I wait (…) in general I wait, I generally do not run immediately if I suddenly get pain (…) Then you first use a cold wrap (…) and then after around three days, if it is not better, then I only go to the doctor.” *

### 3.4. Suggestions for a Better Health Care: The Self-Confident Senior Patient and the Empathic Doctor

An integration of CAM in their health care would be welcomed by the majority of the participants. One participant (FS 22) went even as far as demanding for this integration. Most of the participants would prefer natural compared to chemical products and would appreciate to get substantial and neutral information about CAM from their family doctors. At the same time the patients would need to learn to build their own opinion and be able to discuss this with their doctors. The participants also asked for better trained doctors in CAM and developed some ideas about this for medical schools.

 FS 22: *“[CAM as] a specialty that students have to go to (…) which is part like surgery or internal medicine or ophthalmology or otology.” *

The participants all agreed upon the wish for more empathy in general. The participants asked for more dialogues, more interest, more humanity, and a reliable follow-up care. Also experience, thoroughness, and physical examination were required which would need also more time.

 FS 24: *“I think the doctor should open his ears and listen carefully, what I say (…) I often sensed or felt, man, he is not really here with his mind.” *

Half of the participants suggested more cost coverage by health insurances for CAM and the dialogue between patient and doctor. There was consensus that a physician cannot do everything alone and that cooperation with other physicians and other specialists was important. One discussion group outlined even an ideal clinic with a nurse, a diet assistant, a social worker, a physician specialized in psychosomatics, other specialized physicians, and a family doctor who coordinates all. The family doctor should also do home visits which are important in older age. 

### 3.5. Specific Issues and Worries of the Elders

One male senior (MS 32) expressed his deep worries about possible dependency on care and incapacitation, for example, because of dementia. All participants agreed that the best prophylaxis of dementia is to stay mentally and physically active and flexible including special memory training program, internet, reading newspapers, and doing crossword puzzles. Furthermore plants like ginseng, gingko, and food supplements with fish oil were discussed. However the repeated change of scientific information in regard to dementia led to confusion and doubts.

 MS 32: *“If you deal regularly with the internet, that is said to be good.” *
 FS 11: *“Nor fish oil or anything else, nor memory training, all this wouldn't help.” *

Other sorrows were that elder people are seen by the society as a burden with regard to health costs, the lack of interest of the younger generation, and difficulties in intergenerational understanding. This also influences the interaction with younger physicians.

 FS 24: *“The older you get the quicker your are out of the doctor's office.” *

### 3.6. Gender Aspects

The male seniors expressed more often worries like fear of care dependency and highlighted that being of older age and retired does not mean to be free from stress, pressure to perform, and the fast pace of our time. In contrast to that among the female seniors the image of an old and wise female healer was brought up.

 MS 32: *“One thinks always, that as a pensioner you would not have any stress. (…) [but] smallest causes (…) can raise stress or panic (…) one is insecure (…) when I think of the time I was working, the world could have open up and I would have stayed cool (…) but that changed (…) you get more sensitive (…) that you have to admit.” *
 FS 3: *“I do not know, if one relates more to oneself in higher age. (…) As woman one discovers the healer in oneself and this wisdom.” *

The men were mostly informed about CAM by their wives. Nevertheless they showed interest in CAM and gathered a lot of knowledge about it. Women used more home remedies and were more open to relaxation techniques. Men had a special interest in nutrition, partly embedded in eating traditions. Men and women showed the same interest in exercise.

## 4. Discussion

The results show that CAM therapies are used by most participants of this study and that more integration in health care would be appreciated. Their CAM use originated from knowledge and experience in their childhood at a time when proper access to conventional medicine was limited or unavailable. There are several discontents with current health care provision, which lead to wishes for more empathy, time, and personal contact with their physicians. 

### 4.1. Advantages and Limitations

The use of “World Cafés” is relatively new in qualitative research [9] and was proven to be useful in this study. Small breaks when groups were changing, moving to another table, and getting engaged in different groups created a lively, open, and creative atmosphere and reduced the possibilities of building patterns in group dynamics. Many different perspectives were gathered. In particular the development of new ideas seems to be well supported by the methodology of the World Café. This setting did not apply to the male focus group. There the atmosphere was calm and there were no time limits, which might have helped in creating an atmosphere of trust. Both settings are different but seemed to be useful in discussing the topics of this study because in both settings lively discussions took place and results were not so different. However, the difference between men and women in talking about worries might have been due to the different settings.

A limitation of this study could be the sample. Even though the neighborhood center is open to everybody and seniors come from all over the city and different social classes, one can expect that only interested and already active seniors go there. That could be a reason why there was a tendency towards a positive attitude regarding CAM and an active, self-responsible life in the discussions. Because male seniors where known to attend activities of the neighborhood center less than female seniors, especially men were invited to the “World Café.” Still only women joined. To include men's perspectives a second discussion group was necessary. With the recruited men a “World Café” would not have made sense; therefore a focus group was conducted. The huge interest of women attending the “World Café” and their positive tendency towards CAM relate also to findings of other studies, which showed that women have a bigger interest in CAM than men [3]. According to the socioeconomic factors, about half of the participants had a higher education (Table 1) which is a known factor for a more frequent use of CAM [11]. 

### 4.2. Life Experience: The Connection between Conventional Medicine and CAM

This study showed that according to the participants' conventional medicine and CAM cannot be seen as totally separate entities. To separate conventional medicine and CAM, for example, in areas such as nutrition and exercise, was not possible and even not relevant for the seniors. The academic and modern term CAM was not of interest for them. More relevant for the participants seemed to be the distinction between natural and traditional therapies with personal contact to physicians in contrast to chemical, modern, technical, and impersonal therapies and treatments. 

As shown in the survey of Cheung et al. [2] reasons for using CAM were dealing with various physical and mental problems, prevention of diseases, and personal control over health. Within this context, self-care in the sense of empowerment, self-initiative, and self-responsibility played an important role. In this study the fear of dementia and of dependency on medical care were of high importance. Despite contradictory and confusing statements from science, staying mentally and physically active and flexible was seen as a core to prevent dementia, other diseases, and dependency on medical care. Similar aspects were reported in a qualitative study on elderly suffering from neck pain [12].

Further motives for CAM use in our study were enhancing wellbeing and quality of life and the fear of side effects from conventional medicine. The use of CAM might also be partly seen as a reaction to a fragmented and impersonal conventional medicine and as a bridge to a more natural, human, and holistic medicine. Studies about the motives of younger adults towards CAM use showed similarities in participants' emphasis on the holistic nature and the pragmatism of the approach [13]. In this study, the pragmatic attitude was expressed by using therapies that were known among the participants and were easily accessible. Relating to the findings in several surveys CAM therapies used most often were herbs, nutrition, and relaxation/meditation. However, the importance of physical exercise and the openness towards many different types of movement including yoga and qigong was not seen in the surveys. In our German sample, chiropractic manipulation was not mentioned whereas it was often used in the US according to the surveys [2, 3, 14]. Both could be explained by differences in the health systems: in the US chiropractic is part of CAM whereas it is part of conventional medicine in Germany. Exercise might not have been included in the US surveys because it is not always considered a CAM method, similar to that which was discussed in our study. Methods such as homeopathy and acupuncture were less often used and opinions about its effects varied [11]. Although most of the used methods were known from childhood on, there were an openness and curiosity to new ways of healing, for example, meditation which can also be seen in openness to attend studies in CAM [15–17].

The knowledge of seniors could be seen as a resource and should be further explored. This relates to more reintegration of the wisdom of the elderly in health care [18]. For example, in senior mentoring programs in US medical schools, where seniors mentor medical students, knowledge of the elderly is integrated and enhances the communication between generations [19]. Furthermore to integrate life experiences is one part in the concept of healthy aging [20].

### 4.3. Integration of CAM in Health Care: The Importance of Communication

Corresponding to a survey by Zhang et al. [14] elder people in our study were interested in communicating about CAM with their doctors but still did not always inform them, because of their possible negative reaction. Furthermore they felt insecure about the CAM knowledge of their physicians. In a qualitative study Shelley et al. [21] show that a lack of interest of physicians in knowing about CAM use of their patients also occurs in interactions with younger patients. Other reasons for not informing doctors about CAM use shown in this study is the felt lack of real interest and empathy that is correlated with a lack of time but also possible intergenerational misunderstanding. Nowadays conventional medicine was perceived as mainly technically orientated and having no real personal and physical contact between patient and physician, which might contrast experiences with conventional medicine in childhood and youth [22, 23]. Interactions and side effects of drugs and inappropriate medication in the elderly are a well-known problem [4–7], resulting in increased morbidity and mortality [24]. Having no knowledge of additional CAM use increases the safety problem in a population that uses multiple drugs [2, 25]. 

### 4.4. Suggestions for Medical Care: Empowerment and Empathy

Most of the participants would welcome an integration of CAM into their health care. They would prefer natural over chemical products and would like to get understandable and unbiased information about CAM [14]. They asked for improved education of physicians in CAM and more cost coverage of CAM by the health insurance companies. This correlates with physicians' interest in better education and training opportunities in CAM treatments [26, 27]. 

In general the participants wanted a better contact with their family doctors, more empathic consultations, more physical examinations, more time, more individual-specific care, and reliability including follow-up care. Because the family physician cannot do and know everything, cooperation of different specialists in health care, social care, and CAM was seen as relevant. Participants wanted to get specific advice on self-care options to improve health or treat diseases and preferred shared-decision making. Also in younger adults shared-decision making seems to be an important aspect that motivates patients to visit CAM practitioners [28]. At the same time it was considered very important in this study that the knowledge and experiences gained in their lifetime should be more valued, respected, and appreciated by their physicians. 

## 5. Conclusions 

This qualitative study shows that seniors use self-care strategies including CAM methods which were often learned in childhood and applied by seniors throughout their life in addition to conventional treatment strategies. The modern medical model with its extensive use of technology was critically discussed. Life experiences and desire for one's own initiative can be seen as resources and an integration in health service provision could be useful in creating a sense of empowerment of senior patients, for example, in preventive care.

The participants identified intergenerational conflicts and misunderstandings with their doctors. Therefore qualitative studies investigating intergenerational doctor-patient relationship in CAM use could be of interest for further studies. Thereby the perspective of the senior patients and the doctors would need to be addressed.

## Figures and Tables

**Table 1:** Demographic data of the participants.

Variable	Patients *N* = 21 mean ± sd/*n* (%)
Gender	
Female:	18 (85.7%)
Male:	3 (14.3%)
Age (years)	72.5 ± 4.3
Range:	65–83
Civil status	
Single:	3 (14.3%)
Married:	6 (28.6%)
Divorced:	7 (33.3%)
Widowed:	5 (23.8%)
Graduation	
School <10 years:	5 (23.8%)
School ≥10 years:	15 (71.5%)
No declaration:	1 (4.8%)

**Table 2:** CAM treatment and their frequency.

Category	Frequency *N* = 21 *n* (%)
Food supplements	6 (28.6%)
Phytotherapy, internal	6 (28.6%)
Phytotherapy, external	3 (14.3%)
Wraps	3 (14.3%)
Homeopathy	2 (9.5%)
Hydrotherapy	2 (9.5%)
Relaxation	1 (4.8%)
Exercise	1 (4.8%)
Others (e.g., conventional medicine)	6 (28.6%)

## Abbreviations

CAM: Complementary and alternative medicine
FS *x*: Female senior nr. *x *
MS *x*: Male senior nr. *x*.
